# Mechanistic Modelling of DNA Repair and Cellular Survival Following Radiation-Induced DNA Damage

**DOI:** 10.1038/srep33290

**Published:** 2016-09-14

**Authors:** Stephen J. McMahon, Jan Schuemann, Harald Paganetti, Kevin M. Prise

**Affiliations:** 1 Department of Radiation Oncology, Massachusetts General Hospital, 30 Fruit St, Boston, MA 02114, USA; 2 Centre for Cancer Research and Cell Biology, Queen’s University Belfast, Belfast, BT9 7AE, N. Ireland

## Abstract

Characterising and predicting the effects of ionising radiation on cells remains challenging, with the lack of robust models of the underlying mechanism of radiation responses providing a significant limitation to the development of personalised radiotherapy. In this paper we present a mechanistic model of cellular response to radiation that incorporates the kinetics of different DNA repair processes, the spatial distribution of double strand breaks and the resulting probability and severity of misrepair. This model enables predictions to be made of a range of key biological endpoints (DNA repair kinetics, chromosome aberration and mutation formation, survival) across a range of cell types based on a set of 11 mechanistic fitting parameters that are common across all cells. Applying this model to cellular survival showed its capacity to stratify the radiosensitivity of cells based on aspects of their phenotype and experimental conditions such as cell cycle phase and plating delay (correlation between modelled and observed Mean Inactivation Doses R^2^ > 0.9). By explicitly incorporating underlying mechanistic factors, this model can integrate knowledge from a wide range of biological studies to provide robust predictions and may act as a foundation for future calculations of individualised radiosensitivity.

Following more than a century of investigations into the impact of ionizing radiation on biological systems, radiation is one of the most well-studied genotoxic agents and a key cancer therapy. Much of our modern understanding of genetics and cellular biology has been built, in part, on investigations of irradiated cells and tissues^1^. However, despite this wealth of knowledge, most clinical radiotherapy is still based on the application of the linear-quadratic model, defining survival, *S*, in terms of the dose *D* of radiation received and two empirical fitting parameters *α* and *β*: 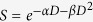. Whilst it is widely acknowledged that this is only an approximation of the true behaviour of the system and considerable debate exists about its wider applicability^2^, this characterisation represents a useful surrogate for tumour control and normal tissue complication and broadly captures the sensitivity of tumours at a population level. As a result, the linear-quadratic model remains a mainstay of clinical practice, for example in the calculation of biologically effective doses (BEDs) to compare the impact of different fractionation schedules^3^.

Population-level characterisations of radiosensitivity are necessarily limited, however, as highlighted by our growing knowledge of cancer’s underlying genetic hetreogeneity^4^. This heterogeneity is reflected in the varied radiation response of tumours–even among those arising in the same sites–as seen in *in vitro* experiments and clinical response^5^. The integration of genetic heterogeneity to provide personalised radiotherapy dose prescriptions could significantly improve treatment outcomes and offer a more rationally informed balance between tumour control and normal tissue toxicity.

This seems particularly attractive given that many of the genetic determinants of radiation response are well understood–processes such as DNA repair and cell-cycle arrest have been extensively studied, both directly with radiation as well as in the wider field of molecular biology. However, the linear-quadratic model is poorly suited to understanding these factors, as its empirical parameters are only indirectly linked to the mechanistic drivers of radiation response, making it difficult to predict quantitatively the impact of a given mutation. This is particularly true when multiple genes are mutated, as occurs commonly in cancer.

One approach to understand this heterogeneity is to use bioinformatics techniques to identify key genes or signatures to stratify patients into different sensitivity groups^6^,^7^. While promising, these approaches typically do not take advantage of our mechanistic understanding of radiation response, and so are often poorly conditioned and require extremely large data sets to generate robust, translatable signatures.

An alternative approach is to incorporate these factors into radiation response models. There has been considerable interest in this area, including numerous analytic models which seek to more explicitly incorporate underlying mechanisms (such as the Lethal-Potentially Lethal lesion or the Repair-Misrepair models^8^,^9^), and the inclusion of additional parameters to reflect conditions such as dose rate (the Lea-Catcheside dose protraction factor), oxygen availability (Oxygen Enhancement Ratio), radiation quality (Relative Biological Effectiveness) and tissue geometry^10^,^11^,^12^,^13^,^14^. In addition, more detailed cell survival models have been developed which seek to more explicitly incorporate these effects in their underlying model structure^15^,^16^. Alongside this, numerous models have been developed of individual pathways such as DNA repair, chromosome aberration formation, apoptosis or intercellular signalling^17^,^18^,^19^,^20^.

However, these models are often limited to focus either on a single pathway or endpoint, and as such cannot incorporate the broad spectrum of response data within the radiation biology literature. In this work, we develop a model which implements high-level characterisations of DNA repair through different pathways, cell cycle effects, and cell death processes to provide a mechanistically defined model of cellular responses to ionising radiation. This model is based on a temporal and spatial model of DNA repair incorporating a Gaussian spatial dependence of Double Strand Break (DSB) misrejoining and varying fidelities of different repair processes. Based on this approach, the model is able to predict cellular responses for multiple endpoints for a range of experimental conditions and cells of different genetic backgrounds, based on a single set of parameters common to all cell lines rather than ad-hoc parameter sets for each cell line. This model provides a framework to incorporate mechanistic genetic knowledge into radiosensitivity predictions and to support the development of personalised radiotherapy models, and demonstrates an approach which may be applied to better understand other clinical techniques in the future.

## Methods

### Model Overview

This model initially focuses on DSB induction and repair following exposure to sparsely ionising X-ray radiation. Because of the low Linear Energy Transfer (LET) of such radiation, resulting DSBs are distributed randomly and uniformly throughout the nucleus. Developments to other radiation types may later draw on nanodosimetric models to generate more accurate spatial DSB distributions^21^.

Repair of these DSBs is modelled in a temporal fashion, taking into account the three key repair processes within cells–Nonhomologous End Joining (NHEJ), Homologous Recombination (HR), and Backup or Microhomology Mediated End Joining (MMEJ). As each break is repaired, it can either be repaired correctly (where the two free ends of a DSB rejoin correctly), or misrejoined, when ends from two distinct DSBs are combined, leading to genetic alterations which can have physiological impacts on the cell. The rate of rejoining between any two free ends is taken to scale with the distance between them as 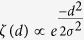 where *ζ* is the rejoining rate, *d* is the separation between ends, and *σ* is a characteristic rejoining range. This rate applies to both ‘correct’ ends (the pair created by a single DSB) as well as all mismatched pairs. Such an approach has been adopted by other researchers in the past to describe spatial effects in radiotherapy^18^,^22^. By considering both the total rate of rejoining and the spatial distribution of breaks, other factors can be calculated such as the rate of inter- and intra-chromosome events and the size of resulting deletions. These rates can then be used to generate predictions of various biological endpoints.

While such a model can readily be implemented in a stochastic Monte Carlo fashion, this is often time-consuming, particularly for rare endpoints, and so analytic solutions were derived for the rates of relevant processes. This analytic model is summarised below, with additional details on its implementation and validation presented in the Supplementary Information, along with a Python implementation.

### DSB Induction and Repair Kinetics

X-ray induced DSBs have been extensively studied, with results suggesting an initial yield of approximately 35 DSBs/Gy in human cells in the G1 phase of the cell cycle^23^. This value scales linearly with the DNA content of the nucleus, e.g. across species or between different phases of the cell cycle^23^. Assuming a human diploid cell contains 6.1 Gbp (Genome Reference Consortium, GRCh38.p6), this gives a rate of 5.738 DSBs/Gy/Gbp, which is taken as a constant.

These breaks are repaired by one of three processes. In normal cells, NHEJ can repair DSBs throughout the cell cycle, and HR becomes available in late S and G2 phases for higher-fidelity repair of complex damage using the replicated sister chromatid. Finally, in cells where NHEJ or HR is inhibited a fall-back process known as Backup or Microhomology-Mediated End Joining (MMEJ) is used, although this pathway is significantly slower and more error-prone^24^.

We model the repair of breaks by each process with independent exponential kinetics with characteristic rates. This gives rise to a three-phase behaviour for DNA repair, with breaks repaired either with fast kinetics (simple breaks, via NHEJ), slow kinetics (complex DSBs, repaired via HR when available and NHEJ if not) and very slow kinetics (breaks repaired via MMEJ due to HR or NHEJ failure). The overall behaviour is then:


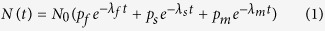


where *N*(*t*) is the total number of DSBs at time *t*, *N*_*0*_ is the initial number of DSBs, *λ*_*x*_ are the three repair rate constants (**F**ast, **S**low, and **M**icrohomology, respectively), and *p*_*x*_ is the probability of a break being repaired by each process.

Rather than directly fitting *p*_*f*_, *p*_*s*_ and *p*_*m*_ for each cell line, these probabilities are calculated on a mechanistic basis based on two fitting parameters. Firstly, breaks are randomly selected to be either simple or complex based on a probability *p*_*c*_. This parameter is related to the quality of the incident radiation, and is taken as a single fitting parameter across all X-ray radiation types. Simple breaks are initially associated with fast repair kinetics, and complex breaks with slow repair kinetics (that is, *p*_*f*_ = (1 − *p*_*c*_), *p*_*s*_ = *p*_*c*_ and *p*_*m*_ = 0 in repair-competent cells).

Next, if a cell has a defect in the preferred repair pathway for a break, a portion of these breaks will fail to be efficiently repaired with probability *p*_*fail*_ and will instead be repaired by MMEJ. For example, if a cell in G2 has a defect in NHEJ, the repair probabilities would become *p*_*f*_ = (1 − *p*_*c*_)(1 − *p*_*fail*_), *p*_*s*_ = *p*_*c*_ and *p*_*m*_ = (1 − *p*_*c*_)*p*_*fail*_. A full tabulation of this approach is presented in the Supplementary Information.

This approach gives a set of five fitting parameters (*λ*_*f*,_ *λ*_*s*,_ *λ*_*m*,_ *p*_*c*_, and *p*_*fail*_) which characterise the rates of repair as a function of time in cells under a range of experimental conditions and genetic backgrounds.

### DSB Repair Fidelity

While DSB repair is a high fidelity process, there remains a significant risk of misrepair leading to mutations and genetic aberrations, which is known to be a spatially-dependent process^18^,^22^. By modelling the behaviour of breaks using the Gaussian rejoining probability outlined above, the fraction of misrepaired breaks can be calculated as a function of the initial number of breaks, *N*_*0*_, and *σ*. For breaks distributed uniformly within a spherical nucleus, it can be shown (see Supporting Information) that the probability of a DSB correctly rejoining is given by


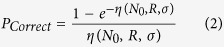


where η is the effective integral misrejoining probability for a single randomly placed DSB within a nucleus of radius *R* with rejoining range *σ* when there are initially *N*_*0*_ other DSBs present within the system. This rejoining rate can be exactly calculated as:


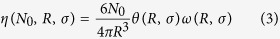


where the first term is the density of free DSB ends within the nucleus, *θ* is the rejoining rate between two randomly placed DSB ends, and *ω* is a correction coefficient to account for skew in the *η* distribution for very small *σ*. These can be calculated as:


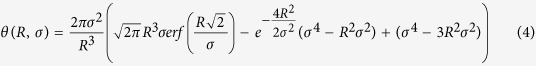



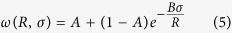


where *A* and *B* are geometric fitting parameters, with values 0.757 and 5.39 respectively, determined by matching this function to the output of a Monte Carlo simulation of break rejoining, as illustrated in the Supplementary Information.

One additional factor in DNA repair is that different processes have different inherent fidelities, even in the absence of misrejoining with other breaks. To reflect this, we associate a base fidelity with NHEJ and MMEJ, such that the probability of correct repair is given by


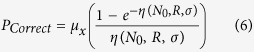


where *μ*_*x*_ is a process-specific fidelity. For homologous recombination, it is assumed that repair always completes successfully, giving *P*_*Correct*_ = 1.

While this provides total misrejoining rates, the severity of these events is governed by the location of the involved DNA ends, and can range from a short deletion to aberrant bridges between chromosomes. Quantifying the rates of these different events is an important factor in modelling the effects of ionizing radiation on DNA^25^.

### Chromosome Aberrations–G1

Chromosome aberrations are large-scale genomic rearrangements between and within chromosomes leading to the deletion of DNA or the formation of aberrant chromosomes (rings, dicentrics) that are unable to segregate properly during mitosis. These events can have a high probability of lethality and are one of the most biologically significant effects of ionising radiation.

Aberrations can be classified as *inter*- or *intra*- chromosome, depending on which chromosomes provided the damaged free ends. As a highly simplified model of chromosome structure, chromosomes are modelled as spherical sub-volumes with radius 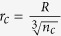, where *n*_*c*_ is the number of chromosomes within the nucleus. This approach neglects variations in chromosome size, shape and packing that would be expected to impact the type and distribution of aberrations. However, as this analysis focuses on average rates across the whole nucleus, the impact of these factors is significantly reduced. When misrepair occurs, the probability of an intra-chromosome event is given by the ratio of the rate of rejoining within the chromosome to that across the whole nucleus, given by:


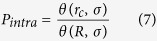


A second important classification is whether the exchange is symmetric or asymmetric. In a symmetric exchange, all chromosomes contain exactly one centromere, while asymmetric exchanges form acentric fragments, dicentrics and other complex rearrangements^25^. Symmetric exchanges are typically non-lethal, while asymmetric events are often incompatible with cell survival.

The symmetry of an exchange is solely determined by whether the DNA ends which rejoin are correctly aligned (i.e. centromere-containing fragment to centromere-free fragment) or not. As the DNA ends are otherwise identical, symmetric and asymmetric exchanges are taken to occur with equal frequency, that is *P*_*asym*_ = 0.5.

Based on these assumptions, the total number of misrepaired DSBs, and corresponding number of dicentrics (asymmetric inter-chromosome) and deletions (asymmetric intra-chromosome) can be calculated as:


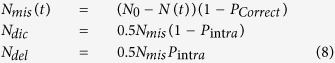


where *N*_*mis*_(*t*) is the number of misrepaired DSBs at time *t*, and the factor 0.5 represents the probability of an asymmetric exchange. Because of the time dependence of this term, formation of dicentrics and deletions is also a time-dependent process, however for brevity this is excluded from subsequent expressions. For the purposes of this analytic formulation, all acute exposures are considered as happening instantly, meaning all *N*_0_ DSBs are created instantly before any repair. More complex patterns incorporating intra-exposure repair can potentially be calculated using Monte Carlo techniques.

For deletions, size is also an important factor. Based on the assumption that the separation of two breaks in base pairs along the chromosome increases monotonically with spatial distance between the free ends, the size of a deletion in base pairs is 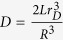, where L is the total length of all chromosomes (in Gbp) and *r*_*D*_ the separation between ends forming the deletion.

The rate of deletions smaller than *D* is given by the rate of misrejoining events over distances shorter than *r*_D_, assuming both events occur within the same chromosome. This is given by


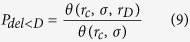


where the generalised form of θ is:


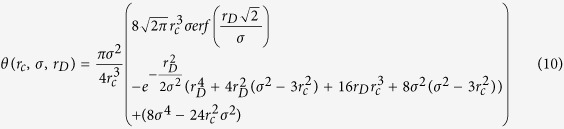


As the visibility and lethality of lesions often requires them to be above a certain size, the number of deletions larger than *D* can be expressed as:


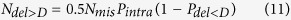


### Mutation Rates

In addition to lethal aberrations, radiation can induce non-lethal mutations where genes are deleted or altered, leading to changes in cellular function. Mutations are typically assayed on single genes whose alteration has significant impacts on cell survival in a particular condition–such as the Hypoxanthine-guanine phosphoribosyl-transferase (*Hprt*) gene^26^. These mutations are typically rare events, and are dependent on the size of the gene under consideration.

Misrepair events can cause mutation in two ways–either partial alterations (breaks within the gene misrejoining) or total deletions (two breaks outside a gene misrepairing to create a deletion which spans the gene).

The number of total deletions for a gene of length *g* can be calculated as:


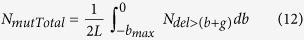


where for each point at a distance *b* base pairs before the start of the gene, the probability of a deletion event occurring which spans the whole gene in consideration is calculated, ranging from the start of the gene (*b* = 0) to *b*_*max*_, the largest deletion which is compatible with cell survival. This is normalised according to the total length of the genome, *L*.

For intra-gene events, the rate can be calculated by assuming that all misrepair events involving a break within the gene are taken to lead to mutations, given by:


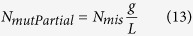


In addition to misrepair events, even joining of the correct DSB ends may lead to short point mutations in a gene due to imperfect repair leading to small insertions or deletions. To reflect this, a point mutation factor, *ν*, is introduced, with the number of point mutations given by:


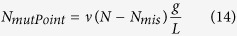


### Chromosome Aberrations–G2

In G1, inter-arm exchanges (intra-chromosome events which span the centromere) do not have increased visibility compared to other intra-chromosome events. However, in G2 these events have a much higher visibility due to the structural changes involving the sister chromatid. The rate of inter-arm aberrations can be estimated by considering the rate at which an aberration spans some or all of the centromere, in a similar way to the deletion of a single gene, above. Thus the inter-arm aberration rate is similarly given by:


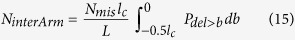


where the centromere is assumed to have negligible size (i.e. *g* = 0). The integral is carried out over the whole average chromosome length *l*_*c*_, as chromosome aberration assays typically do not require the long-term survival of the cell.

### DNA Repair Model Implementation and Fitting

This model has been implemented in Python for a general cell, based on two sets of input values. The first is the set of nine mechanistic fitting parameters-*λ*_*F*_, *λ*_*S*_, *λ*_*M*_, *p*_*c*_, *p*_*fail*_, *σ*, *μ*_*NHEJ*_, *μ*_*MMEJ*_ and *ν*, which are the same across all cell lines. The second set are those specific to a given experiment. These include details of the phenotype of the cells being investigated (its genome size and chromosome number, and any defects in DNA repair processes) and experiment-specific parameters (the dose delivered, the phase the cells were in during irradiation, and the time after irradiation when the endpoint was measured).

Cell-specific values (such as the initial number of DSBs and the proportions of repair by different processes, *p*_*f*_, *p*_*s*_ and *p*_*m*_) are then determined by combining these experimental and mechanistic parameters. From these, observable endpoints such as residual DSBs, numbers of visible and lethal chromosome aberrations, and rates of mutation can be calculated.

To fit these model predictions, a number of data sets were obtained from the literature, including measurements of DNA repair through γH2AX fluorescence^27^,^28^, DNA misrepair rates using PFGE^29^,^30^, chromosome aberration yields^18^,^31^,^32^,^33^,^34^,^35^,^36^,^37^ and mutation rates^38^,^39^,^40^. Values were extracted from published tables or figures along with associated uncertainties. To reflect uncertainties in data extraction and experimental methods, an additional 5% random uncertainty was added to all points. A single simultaneous fit was carried out across all data varying the 9 fitting parameters. By simultaneously fitting to a wide range of endpoints, the model generates a robust, unique parameter set, with reasonable uncertainties and small covariances. This fit was carried out in Python using the SciPy curve_fit routine, using a weighted nonlinear least squares algorithm. The resulting best-fit parameters are presented in Table 1, and these values were used to calculate all of the curves presented below. The model implementation, fitting algorithm, and input data sets are presented in the Supplementary Information, together with the characteristics of cell lines analysed as part of this work.

A number of additional endpoints (yields of sub-types of chromosome aberrations, low dose rate chromosome aberration yields and point mutation rates) were extracted, but not incorporated in the fit, to test underlying model assumptions.

### Survival

Cell survival is one of the key biological endpoints of radiation exposure. In the simplest case, the response of non-cycling cells (e.g. those arrested in G1/G0) is driven by the formation of lethal chromosome aberrations–that is, those which prevent chromosome segregation at mitosis (dicentrics, rings) or those which disrupt cellular function through the loss of genetic material (large deletions). Such effects have been shown to correlate well with Giemsa-stained aberration counts, which are sensitive to deletions greater than 3 Mbp in size^41^.

For non-cycling cells G1 survival can thus be calculated as 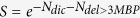 (assuming Poisson-distributed aberrations, a reasonable approximation for X-rays). For cells irradiated in G2, the replicated chromatids mean that either both must see large deletions, or the aberration must be sufficient to prevent successful mitosis. As a result, the survival rate is estimated as 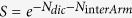. This neglects the case where cells see multiple lethal deletions across chromatids passed down to both daughter cells, but this is relatively rare at clinical doses.

Two other effects are significant in cycling cells–mitotic death and radiation-induced arrest. Mitotic death is triggered when cells undergo mitosis with unrepaired DSBs–either due to newly formed breaks^42^, or escaping the G2 DNA damage checkpoint (which is known to occur when fewer than 20 DSBs remain^43^). The exact mechanisms of mitotic death are not fully understood, but experimental evidence^44^ indicates that these cells die with approximately exponential kinetics in dose, with similar rates across cell lines. Thus, the probability of surviving mitosis is modelled as a single exponential, 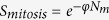, where N_m_ is the number of DSBs present in mitosis and *φ* is a rate constant shared across all cells.

Radiation-induced arrests are complex processes, and are triggered by a range of factors. While they are induced throughout the cell cycle in response to initial damage, they are typically transient once DNA repair is completed. However, they have an important impact on *in vitro* survival in G1, where arrest can be followed by long-term senescence or apoptosis. Quantification of the relative contribution of apoptosis and senescence to survival remains challenging^45^, and even a partial model of these processes is outside the scope of this work. Consequently, a highly simplified model of G1 arrest and apoptosis is considered here. Cells which are cycling and have a functional G1 arrest have a probability of successfully escaping G1 of 
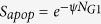
, where N_G1_ is the number of DSBs induced in G1 and *ψ* is again common across all cells. While simple, this is broadly in line with observed kinetics of escape from G1 for irradiated cells^46^. For cells that are not sensitive to these processes (those not cycling, in different phases, or with a mutation in a gene essential to this process such as p53) this event is not triggered.

As for the DNA repair model, survival data was extracted from the literature to validate the model for a range of hamster and human cell lines, different cell cycle phases, different genetic backgrounds and experimental conditions^23^,^27^,^41^,^47^,^48^,^49^,^50^,^51^. To characterise *φ* and *ψ*, a limited fit was carried out to these survival data, taking parameters from the DNA model as fixed inputs to reduce the impact of the relatively low reproducibility of clonogenic survival data.

## Results

### DNA Repair Kinetics

Measurements of DNA repair using γH2AX foci were obtained for mouse and human cell lines with a range of repair defects, irradiated in G1 and G2^27^,^28^. These papers were selected as they represent systematic studies of cell lines with similar origins (human or mouse fibroblasts) with known mutations in individual genes in key DNA repair pathways. It is important to note that there is significant inter-laboratory variability in γH2AX foci counts, even for nominally identical experiments. However, within a single site, relative foci yields are consistent^52^. As a result, for each paper under consideration a single scaling factor was applied across all reported foci counts, to account for variations in staining and counting procedures between groups by normalising each set of results to the model’s predicted total yield of DSBs. These scaling factors were 1.70 and 1.02 for Kühne *et al*.^27^ and Beucher *et al*.^28^, respectively. In addition, observation times were offset by 7.5 minutes to account for the known lag between DSB creation and foci appearance^53^.

**Figure 1:**
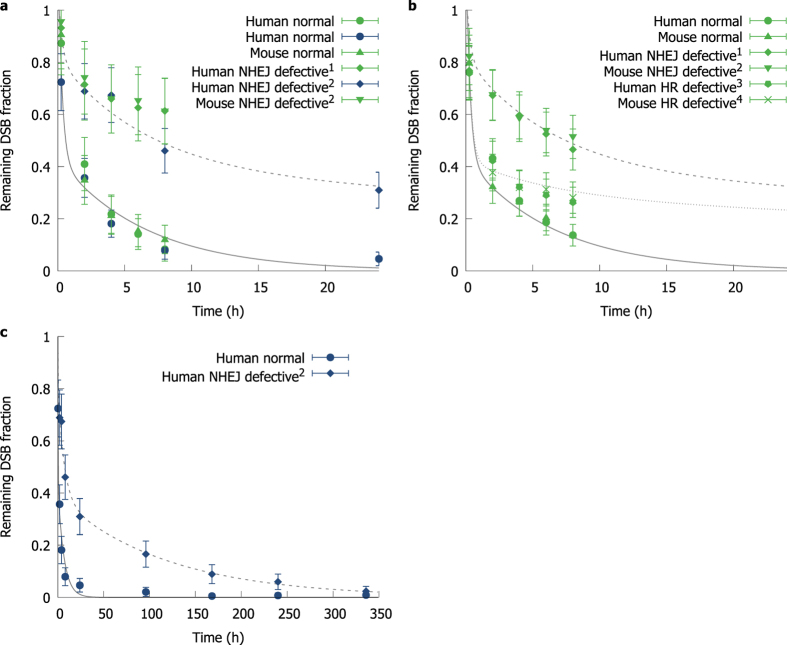
Comparison of modelled DNA repair curves (lines) and experimental data (points) for DNA repair, derived from γH2AX foci measurements. Models were applied to human or mouse cell lines, either with competent repair (solid line) or defective repair (NHEJ defective: dashed line; HR defective: dotted line, not visible in G1). Responses were modelled for G1 (left) and G2 (right), for short and long times (top, bottom respectively). Point types represent different cell lines and defects, and are coloured according to source publication (green Beucher *et al*.^28^, blue Kühne *et al*.^27^). Mutations represented are: 1: XLF mutant, 2: DNA Lig-IV mutant; 3: BRCA2 mutant; 4: Rad54 mutant.

The model’s predictions for these cells are compared to experimental observations in Fig. 1, normalised to the initial number of foci predicted at *t * = *0*. It can be seen that at time-points ranging from 15 minutes to 300 hours, the model agrees well with the overall rates of repair in different phases of the cell cycle for both human and mouse cell lines. As these lines also represent different repair defects, this also enables the determination of unique rate constants for each of the classes of DNA repair, the rate at which complex damage is formed, and the probability of repair failure in DNA repair defective cells.

While such a multi-exponential approach is well established in the analysis of DNA repair kinetics, by combining it with the misrepair models it can provide key constraints on parameters (such as the rate of repair failure in different mutant cell lines) which cannot be directly assessed from misrepair measurements alone. Thus, by combining these different experimental data points, better links can be drawn between fundamental mechanisms.

### Misrepair

**Figure 2:**
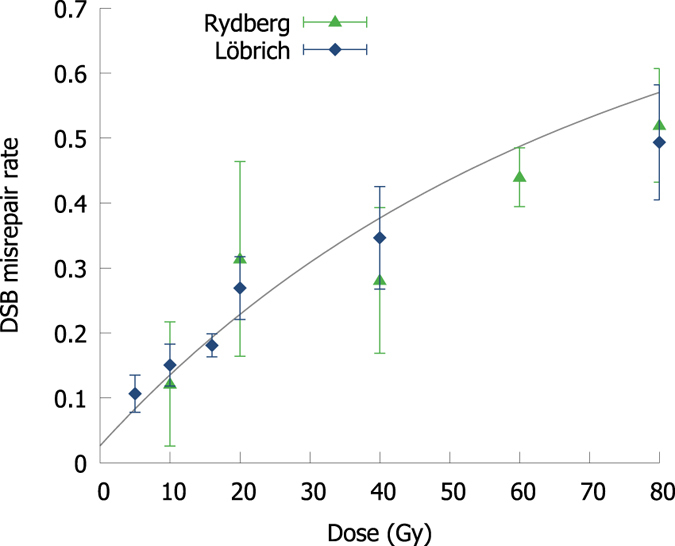
Comparison of modelled rate of misrepair to measurements of incorrectly rejoined DNA fragments via PFGE in normal human cells (From Löbrich *et al*., Rydberg *et al*.^29^,^30^). Although uncertainties in these measurements are large, good correlation is seen over a wide range of doses.

Although almost all DSBs are repaired after long time periods, the fidelity of this repair is not guaranteed, and misrepair is one of the key drivers of radiation-induced cell killing. Pulsed Field Gel Electrophoresis measurements of the correct rejoining of particular sections of DNA have been obtained to assess the overall rate of misrepair in irradiated cells^29^,^30^ and converted to misrepair rates per DSB. These observations were compared to the model’s predicted rates of misrejoining as a function of dose, as illustrated in Fig. 2, showing good agreement at doses from 5 to 80 Gy, although the uncertainties on many measured points are considerable. This correlation indicates that the overall misrepair model is reasonable, scales well at higher doses, and provides parameter constraints for applications to clinically relevant endpoints at lower doses.

### Chromosome Aberrations

There are a number of different techniques to quantify chromosome aberrations, including techniques such as Giemsa staining of chromosomes at mitosis, or Fluorescence *In Situ* Hybridisation (FISH) which can be used to stain specific chromosomes, or in the case of M-FISH^54^ to uniquely stain chromosomes or chromosome sub-regions. These techniques have different resolutions and are able to distinguish different events (for example, symmetric exchanges are frequently not visible under Giemsa staining), making it difficult to make direct comparisons between the results of different studies.

**Figure 3:**
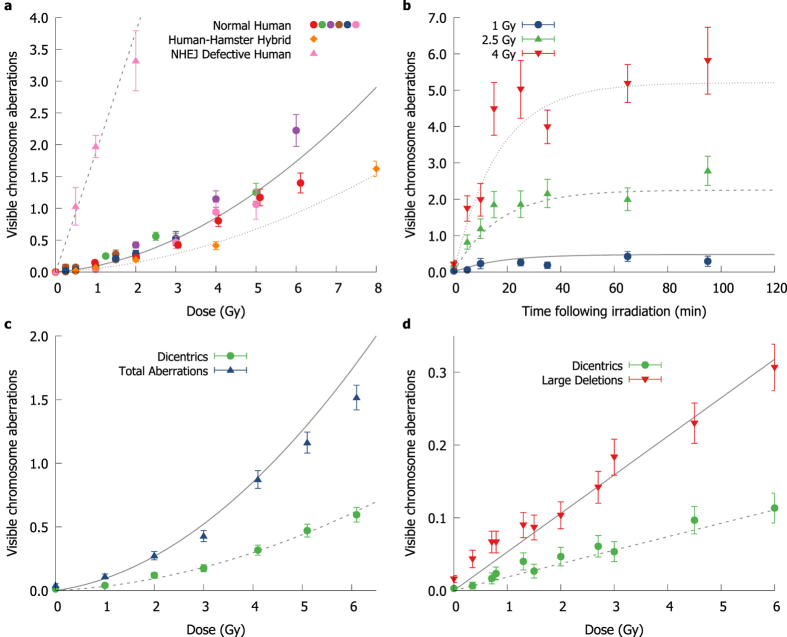
Comparison of experimental measurements and model predictions of chromosome aberrations produced per cell. (**a**) Comparison of overall aberration rates as a function of dose in normal human cells (circles, solid line), hamster-human hybrid cells (diamond, dotted line) and human cells with defective NHEJ (triangles, dashed line), measured through Giemsa staining of cells irradiated in G1^18^,^22^,^31^,^33^,^34^,^36^,^37^. Points are coloured according to source publication. Good agreement is seen across a range of clinically relevant doses, although there is significant inter-experimental heterogeneity between different reports. (**b)** Comparison of aberration yields as a function of time for different doses delivered to cells in G2, assessed through premature chromosome condensation in normal human fibroblasts^32^. The two bottom panels present data not included in overall fitting, analysed to assess model robustness. (**c)** Comparison of yields of dicentrics (dashed line) and total aberrations (solid line) from Cornforth *et al*.^37^. Although dicentrics data was not included in the fit, their proportion is still accurately predicted. (**d)** Induction of chromosome aberrations of different classes exposed at an extremely low dose rate (<10 cGy/h)^37^, where intra-chromosome recombination is negligible. While only acute exposures were included in fitting parameter set, once again good agreement is seen.

In this work, total numbers of chromosome aberrations as a function of dose were obtained from the literature for cells irradiated in G1 and Giemsa-stained at first mitosis^18^,^31^,^32^,^33^,^34^,^35^,^36^,^37^. This assay is sensitive to the formation of dicentrics, large deletions, and rings, and so these observations were compared to model predictions for total yields of dicentrics (N_dic_) and large deletions (*N*_*del>3Mbp*_), as plotted in Fig. 3a. Although there is considerable inter-experimental variation between studies, the overall trend is well reflected across the range of doses considered here. In addition, good correlation is seen for cells with a NHEJ defect, and in human-hamster hybrid cells with a differing genome size and chromosome number.

A similar approach can be applied in the G2 phase of the cell cycle, to aberrations measured through the use of premature chromatin condensation (PCC)^32^. As this technique can measure aberration yields at specific time-points, the kinetics of aberration formation can be quantified. In G2, in addition to the inter-chromosome and large deletion events visible in G1, inter-arm events (*N*_*interarm*_) are also visible, due to the presence of the sister chromatid. Figure 3b compares the observations and model predictions as a function of time. Good agreement can be seen, demonstrating the link between DNA repair kinetics and misrepair, as well as the incorporation of the increased visibility of aberrations in G2 and the increased fidelity of repair by homologous recombination.

While model parameters were determined based on the total yield of aberrations following acute radiation exposures, some other endpoints have also been considered to test model assumptions. Specifically, the relative yield of different aberration types (dicentrics vs deletions) and the yield of aberrations at very low dose rates were compared to model predictions, without being included in the model fitting data. As can be seen in Fig. 3c,d, the model broadly reproduces the total yield of dicentrics as a function of total aberrations, both for acute exposures (3c) and exposures at extremely low dose rates (3d, dose rate <0.1 Gy h^−1^) where multiple-DSB interactions are extremely rare. For the low dose-rate analysis, this is achieved by setting *η* = 0 when calculating repair kinetics, preventing any misrepair resulting from multiple DSBs rejoining.

These results indicate that the simple model of non-overlapping chromosomes applied here does not dramatically impact on the model’s applicability when considering whole-cell rates of genetic aberrations, and that its predicted estimates for the base fidelities of DNA repair are also reasonable.

### Mutations

**Figure 4:**
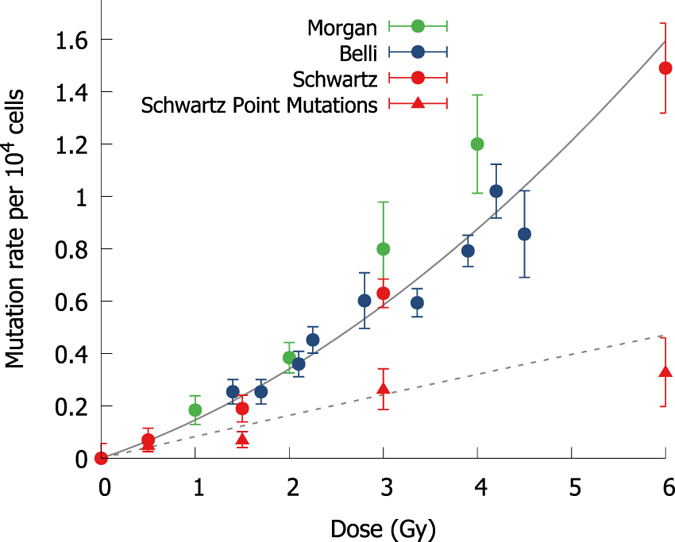
Comparison of modelled yields of mutation in the HPRT gene for Chinese Hamster cells irradiated in G1 (circles, solid line)^38^,^39^,^40^, and the fraction of point mutations (triangles, dashed line)^40^. Points are coloured according to source publication. Overall yield of significant gene damage is accurately reflected, as is the yield of small point mutations, which was not included in fitting data set.

Measurements of the rate of induction of mutation in the *Hprt* gene in Chinese Hamster cells were obtained from the literature^38^,^39^,^40^ and compared to model predictions, as illustrated in Fig. 4. As in the chromosome aberration analysis, these comparisons were made both for the total rate of mutations, which was included in the fitting data set, as well as for the rate of point mutations which was excluded from the fitting data. Good agreement is seen, indicating that the DNA repair model is also applicable to considering the impact of misrepair on specific genetic sub-regions.

### Survival

**Figure 5:**
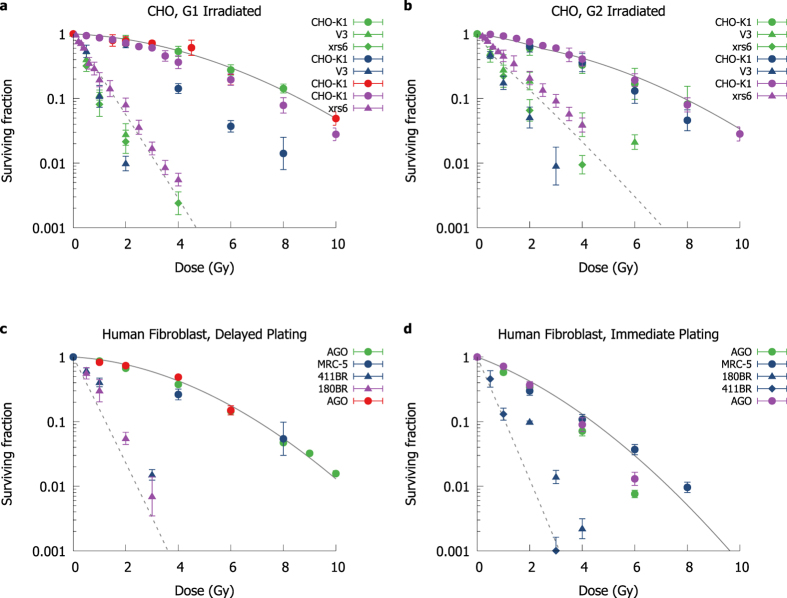
Comparison of modelled cell survival and experimental measurements for cells of different genetic backgrounds in different conditions. (**a**) Survival in Chinese Hamster cells irradiated in G1, either repair competent (CHO-K1, circles) or with defective NHEJ (triangles, diamonds). Curves are plotted for two modelled cell lines, either CHO cells irradiated in G1 with fully competent repair (solid line) or with defective NHEJ (dashed line). (**b**) Survival in CHO cells irradiated in G2, again either repair competent or with defective NHEJ (points as in top left), compared to model predictions (lines). (**c**) Survival in human fibroblast lines irradiated in G1, and held in G1 for over 24 hours before plating for survival assay to prevent G1 cell cycle arrest and apoptosis. (**d**) Survival in human fibroblast lines irradiated in G1 and immediately plated for survival. In lower panels, both normal (AGO-1522, MRC-5; circles) and NHEJ defective (180BR, 411BR; triangles, diamonds) lines are considered, compared to model predictions for either normal (solid line) or NHEJ defective (dashed line) cells, under similar plating conditions. Points are coloured according to source publication. Good agreement is obtained in panels on the left solely from the DNA repair model, as no parameters from the survival fit are needed to predict responses in these conditions. Panels on the right incorporate mitotic death and apoptosis, as described in the main text, to explain these additional conditions. Human data from^27^,^37^,^49^,^50^, hamster data from^23^,^47^,^48^,^51^.

As described above, by considering an exponential dependence between aberration yield and cell survival, predictions of survival can be generated from the DNA repair model for non-cycling cells. These predictions were compared to data from a series of hamster and human cell lines^23^,^27^,^41^,^47^,^48^,^49^,^50^,^51^, selected for irradiations in conditions where there was little sensitivity to cycling effects. For human fibroblast cells, this includes cells irradiated and held in G1 for more than 24 hours before being plated for survival quantification. CHO cells were irradiated in G1, but have a defective G1 checkpoint^55^ and so were not prevented from entering S phase and do not undergo long-term arrest or apoptosis at the G1/S checkpoint. Comparison of model predictions and experimental observations are shown for these cells in Fig. 5a,c. Despite no additional fitting parameters being used, the mechanistic DNA model produces accurate predictions of radiation sensitivity for both repair-competent and NHEJ-defective cells (solid and dashed lines respectively).

In cycling cells, two main additional pathways contribute to cell death–mitotic catastrophe, and active cell cycle arrest followed by senescence or apoptosis. As described above, both of these have been incorporated in the current approach as a simple exponential death process in the current model based on initial yields of DSBs, with the fit parameters (*φ* and *ψ*) presented in Table 1.

**Figure 6:**
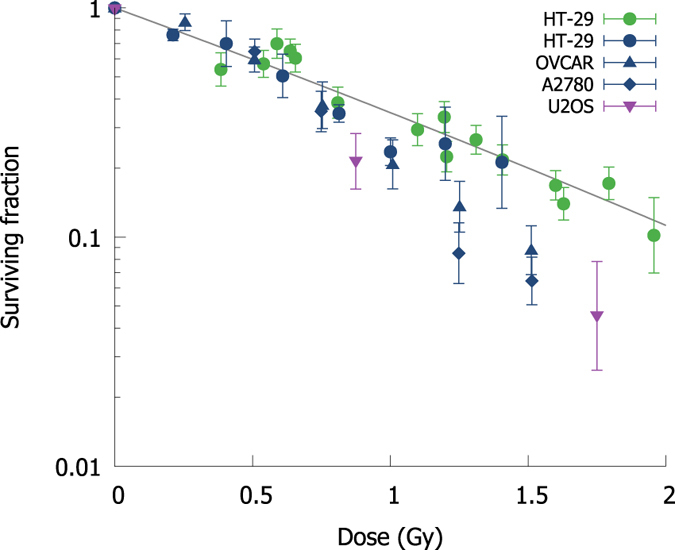
Comparison of modelled cell survival and experimental observation for cells irradiated in mitosis. Symbols correspond to different cell lines, and are coloured according to source publication. For these highly-sensitive cells, death is dominated by mitotic catastrophe following unrepaired DSBs generated during mitosis. Data from^44^,^56^,^57^.

Data for cells irradiated in mitosis was taken from a series of papers^44^,^56^,^57^ and is shown in Fig. 6, while CHO cells irradiated in G2 and human fibroblasts irradiated while cycling in G1 are shown in Fig. 5b,d. Overall trends are well described in all cases, reflecting numerous well-known aspects of radiation response, such as the greater sensitivity of cycling cells to radiation, and the increased radiation resistance of NHEJ defective cells irradiated in G2. Significant inter-experiment variation persists however, even on similar endpoints, because of differences in mutant populations, varying cell cycle selections, plating techniques, and other subtly different experimental conditions which are not currently explicitly incorporated in this model (e.g. in Fig. 5, one CHO cell line shows survival an order of magnitude lower at high doses, perhaps due to the use of serum starvation to synchronise these cells). This suggests that it is important not to over-fit to a particular set of experimental observations.

**Figure 7:**
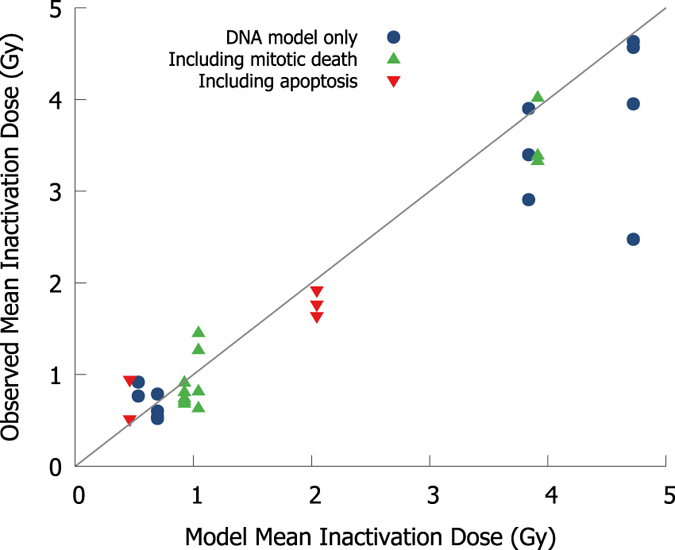
Stratification of cell sensitivity by model predictions, for mean inactivation dose (MID, points), compared to equality (solid line). Cells with identical genetic backgrounds and exposure conditions have equal model MIDs (X-axis), but a sometimes large spread in observed MID (Y-axis). However, despite this the model effectively stratifies across a broad range of radiation sensitivities, within experimental variation between reports on similar cell lines. Model cells tend to be more radiation-resistant than experimental observations (best-fit slope 0.91) which suggests some modes of cell death remain unaccounted for. However, the overall correlation remains high (R^2^ = 0.96), indicating good predictive power.

Despite this, the model still reproduces the behaviour of cell lines well across a range of conditions without cell-line specific fitting parameters. To quantify the model’s ability to stratify radiation sensitivity, Mean Inactivation Doses (MID) were calculated for each data set and for the corresponding model prediction, and compared in Fig. 7. The model effectively stratifies the sensitivity of different cell lines, with a high correlation coefficient (R^2^ = 0.91; 0.96 if divergent CHO line is excluded). In most cases the model slightly over-estimates the MID, suggesting that some death pathways are not yet fully accounted for. Thus while exact quantification of, for example, *α/β* ratios may prove challenging for a specific experimental condition, the model still has the potential to make useful predictions about overall sensitivity.

## Discussion

A primary driver in modern medicine is the movement towards treatment personalisation for a patient’s unique disease, to derive maximum clinical benefit from new or established techniques. While radiotherapy has long been personalised based on geometric factors, tailoring of treatment dose and fractionation remains in its infancy. A key limiting factor is the lack of models with which to predict the sensitivity of an individual’s cancer. This is a particularly pressing need as our understanding of key pathways in radiation response has improved significantly in recent years, supported by molecular and cellular biology experiments as well as improved models of key pathways, offering a wealth of information which could be incorporated into clinical decision-making.

We have developed a general framework of cellular radiation response characterised using cellular phenotypic characteristics that can produce predictions of a range of endpoints including DNA repair, genetic aberration, and cellular survival. While other models have sought to make use of similar mechanistic approaches to radiation response^16^,^17^,^18^,^35^,^58^,^59^, we believe this is the first such model to explicitly incorporate this range of underlying mechanistic processes and endpoints together with cell survival in a single model.

This approach has several benefits. Firstly, by developing a single model which incorporates a range of endpoints, data from different experiments can be combined to inform and constrain model parameters in a way which is not possible when considering empirical fitting functions. For example, this can be seen in the combination of H2AX kinetic and aberration data to characterise how repair processes fail, or the use of a single model to describe both mutation and aberration formation using a single set of fitting parameters, without the need to introduce an empirical scaling factor linking them. Thus while the model incorporates 11 parameters–9 characterising DNA repair, and 2 cell death rates-the fit was carried out across nearly 200 experimental measurements across multiple endpoints, numerous cellular phenotypes, and varying experimental conditions. As a result, the optimum parameter set is very stable and reproducible, with small confidence intervals on most parameters and a limited covariance between parameters (see Supplementary Information). Future developments which make use of additional mechanistic measurements (such as rates of senescence or apoptosis) may enable a further refinement of these parameters.

Another benefit is that in addition to the endpoints explicitly fit here, many established aspects of radiation biology emerge from this model. For example, while the majority of experiments analysed in this work consider single fraction high dose-rate exposures, the model can be naturally extended to prolonged exposures or multi-fraction treatments by incorporating the induction of DSBs in a time-dependent manner, applying the same rules as used in the acute exposure case. Additionally, the current model focuses on X-ray exposures which are assumed to induce DSBs uniformly and randomly throughout the cell nucleus, neglecting any dependence on the linear energy transfer (LET). While this is a reasonable approximation, there is known to be some variation in biological effectiveness for X-rays. Higher LET radiations such as protons or heavier ions can diverge significantly from this approximation, inducing heterogeneously distributed DSBs with differing dose dependence. Incorporation of models to provide radiation-quality dependent DSB information may provide additional insights and the possibility of extending the model to applications such as the calculation of RBE^15^,^21^.

However, significant developments are required before this model can be applied in a general clinical setting. As the model currently focuses on *in vitro* responses, factors such as cellular heterogeneity and oxygen availability are not incorporated, despite being known to significantly impact clinical radiation responses. One approach to address this limitation would be the integration of the model into a 3D agent-based system^60^,^61^. In this approach individual cells are simulated within a three-dimensional tumour volume, which enables the incorporation of both cellular heterogeneity as well as spatially dependent variables such as oxygenation level in a spatially realistic tumour model.

The delivery of personalised radiotherapy will also depend on the development of more refined models of the genetic drivers of radiation response processes. In the current work much of the detail of underlying genetic mechanisms is not considered, with a single model applied across all species and pathways treated either as fully functional or fully defective. In reality, cellular processes depend in a complex fashion on a range of interconnected genes, all of which can have different impacts and may differ across species. The differential impact of genes can be seen in the small differences in sensitivity between xrs6 and V3 cells in Fig. 5. While both cells are treated as identically NHEJ defective, in reality their mutations (in Ku80 and DNA-PKcs respectively) have slightly different impacts on DNA repair or radiosensitivity.

While the current approach is sufficient to demonstrate the viability of the model, explicitly incorporating models of the underlying genetic pathways driving these effects will enable more granular models of the impact of tumour genetics. In this way, rather than population-level models of pathway failure, quantitative estimation of the degree of disruption can be generated for an individual tumour’s genetic information, rather than simply classifying a cell as, for example, NHEJ defective or competent.

By applying this approach to a tumour’s genetic information across all pathways involved in radiation response, an individualised model of the availability of these processes can be generated (e.g. individual values of *p*_*f*_, *p*_*s*_ and *p*_*m*_), which can be combined with the mechanistic parameters obtained in this work to provide personalised predictions of radiosensitivity. As the acquisition of tumour genotypes is becoming increasingly affordable and part of the standard of care for many patients, this offers a natural complement to the growing personalisation in other aspects of cancer therapy. If clinically validated this approach may also be extensible to other clinical applications, beginning with other genotoxic agents (e.g. cisplatin, doxorubicin) whose effects are driven primarily by DNA damage, but also potentially to other agents whose fundamental mechanisms of action are sufficiently well known.

In conclusion, we have developed a mechanistic model of radiation response incorporating a range of key radiation response pathways and clinically relevant endpoints. This model has been shown to accurately describe responses for a range of scales and endpoints with a set of mechanistic fitting parameters which are common across all cell lines, enabling the integration of knowledge from a range of experimental measurements. This model has the potential to offer a foundation for the development of a novel tool to deliver individualised predictions of radiosensitivity in the future.

## Additional Information

**How to cite this article**: McMahon, S. J. *et al*. Mechanistic Modelling of DNA Repair and Cellular Survival Following Radiation-Induced DNA Damage. *Sci. Rep*. **6**, 33290; doi: 10.1038/srep33290 (2016).

## Supplementary Material

Supplementary Information

Supplementary Dataset 1

## Figures and Tables

**Table 1:** List of fitting parameters used in this work, and best fit parameter values from model.

Parameter	Meaning	Value	
DNA Model Parameters			
	DNA Damage Yield	5.738	DSB/Gy/Gbp
*λ*_*F*_	Fast Repair Coefficient	3.6 ± 0.6	hours^−1^
*λ*_*S*_	Slow Repair Coefficient	0.15 ± 0.02	hours^−1^
*λ*_*M*_	MMEJ Repair Coefficient	0.0084 ± 0.0015	hours^−1^
*p*_*c*_	Complex break probability	0.42 ± 0.03	
*p*_*f*_	Repair Failure Probability	0.67 ± 0.09	
*σ*	Misrejoin range	0.0428 ± 0.0005	R_nuc_
*μ*_*NHEJ*_	NHEJ Fidelity	0.985 ± 0.002	
*μ*_*MMEJ*_	MMEJ Fidelity	0.465 ± 0.05	
*ν*	Point Mutation Rate	0.044 ± 0.005	
**Survival Model Parameters**			
*ψ*	Mitosis Sensitivity	0.014 ± 0.002	break^−1^
*φ*	Apoptosis Sensitivity	0.0085 ± 0.001	break^−1^

DNA damage yield is taken as a fixed value from the literature, while other values are presented as best fit parameters ± one standard deviation fitting uncertainty.
