# Supplementary material for: Mechanistic Modelling of DNA Repair and Cellular Survival Following Radiation-Induced DNA Damage
Source: Sci Rep. 2016 Sep 14;6:33290. doi: 10.1038/srep33290 (PMC5022028; doi:10.1038/srep33290)
Supplement: Supplementary Information [file srep33290-s1.pdf]

Mechanistic Modelling of DNA Repair and Cellular Survival Following Radiation-Induced Damage

Stephen J McMahon, Jan Schuemann, Harald Paganetti, Kevin M Prise

Supplementary Information

DNA Repair Kinetics

As described in the methods, the overall kinetics of DNA repair can be characterised as a sum of three independent processes, given by:

$$N(t) = N_0\left(p_f e^{-\lambda_f t} + p_s e^{-\lambda_s t} + p_m e^{-\lambda_m t}\right)$$

Where λ_x are the repair rate constants for each class of damage, and p_x are the fractions of DSBs repaired by each process, out of an initial total of N_0. While similar approaches are commonly used to characterise DNA repair kinetics in different cell lines, these often involve the use of ad-hoc fitting parameters on a cell line by cell line basis. As an alternative approach in this work, a fit was carried out based on DNA repair processes, allowing for more general predictions to be made.

This approach takes place in two steps – firstly, breaks are assigned as either ‘simple’ or ‘complex’, based on a probability p_c. Simple breaks are readily available for repair and so are resolved with fast kinetics, while complex breaks require additional processing before they can be repaired, and are repaired with slow kinetics. This probability p_c is likely a function of both the quality of the incident radiation and the packing of DNA within the cell nucleus. As all irradiations considered within this work are sparsely ionising X-rays, p_c is taken as a single constant fitting parameter in this work.

For repair competent cells, this then gives relative proportions of repair by different processes as $p_f = (1 - p_c)$, $p_s = p_c$ and $p_m = 0$. However, if cells have defects in DNA repair processes, some of these attempts to repair breaks will fail, and be repaired with extremely slow kinetics by MMEJ processes.

The impact of these effects then depends on the cell cycle phase and the particular defect within the cell. Specifically, NHEJ is associated with fast repair throughout all phases of the cell cycle, while HR is associated with slow repair in the G2 phase. If a process is defective, then breaks which would normally be repaired by that process fail and are instead repaired by MMEJ

Cell Type	Repair Competent	NHEJ Defective	HR Defective	
Phase	*All*	*All*	*G1*	*G2*
Probabilities	$p_f = (1 - p_c)$<br>$p_s = p_c$<br>$p_m = 0$	$p_f = (1 - p_c)(1 - p_{fail})$<br>$p_s = p_c$<br>$p_m = (1 - p_c)p_{fail}$	$p_f = (1 - p_c)$<br>$p_s = p_c$<br>$p_m = 0$	$p_f = (1 - p_c)$<br>$p_s = p_c(1 - p_{fail})$<br>$p_m = p_c p_{fail}$

Table S1 Rates of different types of repair as a function of DNA repair defect and cell cycle phase, in terms of damage complexity and repair failure probabilities

with a probability p_{fail}, and so are repaired with extremely slow kinetics. A tabulation of the resulting possibilities for different repair kinetics considered in this work is shown in Table S1. It should be noted that this is necessarily a simplification, as there exist many mutations which may lead to defects of different sizes in repair kinetics, rather than the binary process discussed here, but this model is an adequate approximation for those cells with significant repair defects.

DSB Repair Fidelity – Single Break

In this work, we model DSB repair as a spatially-dependent process, where free ends rejoin with a rate given by $\zeta(d) \propto e^{\frac{-d^2}{2\sigma^2}}$, where d is the separation between break ends and σ is a characteristic rejoining rate. This rate applies to both the correct matching end (i.e. the other paired free end created by the same DSB) as well as incorrect matching with free ends from other DSBs. This model favours correct rejoining as the separation of free DNA ends created by a single DSB is small ($d \approx 0$), but the probability of misrepair increases as the number of additional DSBs increases.

If a single free end from a randomly selected DSB at a position **r** within the nucleus is repaired, the probability that it repairs with the corresponding free end from the same DSB when there are N_0 other DSBs is given by:

$$\frac{\zeta(0)}{\zeta(0) + \sum_{i=1}^{N} 2\zeta(d_i)} = \frac{1}{1 + 2\sum_{i=1}^{N} e^{\frac{-d_i^2}{2\sigma^2}}}$$

Where $\zeta(0)$ is the rate of rejoining with the correct free end to which the separation is small, and the interaction rate with a DSB at distance d_i, $\zeta(d_i)$, is multiplied by 2 to reflect the two free ends created by each break. As $e^{\frac{-d^2}{2\sigma^2}}$ goes to 1 when d is small then the proportionality constant can be factored out, giving the equation on the right. The probability of correct DSB

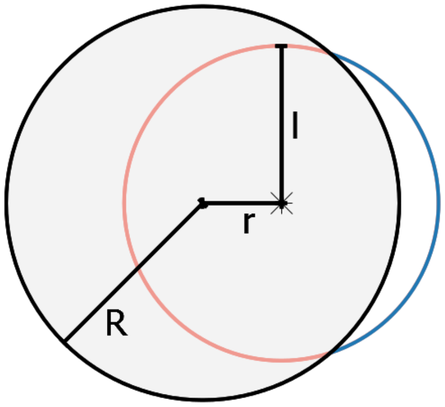

Figure S1. Schematic illustration of rejoining. For a single DSB (cross) at a distance r from the centre of a nucleus of radius R (shaded area), this break would have the same recombination rates for all potential breaks occurring within a shell at distance l. However, a portion of this shell (blue) is outside the nucleus, so only the volume within the nucleus (red) contains DSBs and contributes to the total rejoining rate.

end matching and correct repair thus depends on the sum of the Gaussian term $e^{\frac{-d^2}{2\sigma^2}}$ across all of the DSBs within the nucleus. While this can be readily implemented in a Monte Carlo model to sample overall behaviours, an analytic expression for this rate is valuable, as it makes subsequent calculations significantly more efficient.

DSB Repair Fidelity – Average Break

For a single DSB placed within a cell, the average value of this Gaussian sum can be expressed as:

$$\zeta_{tot} = \int_0^\infty N(l) e^{-\left(\frac{l^2}{2\sigma^2}\right)} dl$$

where $N(l)$ is the expected number of DSBs in a thin shell of thickness dl at a distance l from the DSB under consideration. For a cell with N_0 DSBs randomly distributed throughout the nucleus (such as following X-ray exposure), these DSBs have a density of $\rho = \frac{N_0}{4\pi R^3}$. If the nucleus was of infinite volume at this density, this would give $N(l) = 4\pi\rho t^2$ and the above could be easily integrated to give $\zeta_{tot} = 2\rho\sigma^3\sqrt{2\pi^3}$.

However, because of the finite extent of the cell nucleus, some or all of the points at a distance l may lie outside the nucleus (schematically illustrated in Supplementary Figure 1). This means that $N(l) = \rho V_o(R, r, l)$ where V_o is the volume of overlap between a thin shell of radius l with a sphere of radius R, where the spacing between their centres is r.

The overlap of two spheres of radius R and l at a separation of r can be expressed in three regions. When $R - r > l$, the shell is entirely inside the nucleus and the sphere overlap is given by the full volume, $\frac{4}{3}\pi l^3$. When $R + r < l$, the shell is entirely outside the nucleus, and the shell overlap is 0. The intermediate case, for partially overlapping spheres, is a standard result and given by:

$$V_O = \frac{\pi(R + r - l)^2(l^2 + 2lr - 3r^2 + 2lR + 6rR - 3R^2)}{12l^2}$$

for $R - r < l < R + r$. To obtain the volume of a shell at a given thickness, the sphere overlap can be differentiated with respect to l. This gives a shell volume $4\pi l^2$ for $R - r > l$, and a value of

$$\frac{dV_O}{dl} = \frac{\pi l}{r}(2rl - l^2 - r^2 + R^2)$$

for the partial overlap case when $R - r < l < R + r$.

To simplify the subsequent integration, these values can then be expressed as a fraction of the total shell volume which lies within the nucleus. For shells entirely within the nucleus, this value is 1, and for the partial overlap case is:

$$V_f = \frac{dV_O}{dl} / V_{shell} = \frac{\pi l(2rl - l^2 - r^2 + R^2)}{r4\pi l^2} = \frac{2rl - l^2 - r^2 + R^2}{4lr}$$

If we wish to then determine the rejoining rate between two randomly placed DSB ends, over all possible DSB radial positions r and over the full nuclear volume, we can calculate:

$$\Theta = \frac{1}{\frac{4}{3}\pi R^3} \int_0^R \int_0^{2R} V_f \, e^{-\left(\frac{l^2}{2\sigma^2}\right)} 4\pi l^2 4\pi r^2 dl dr$$

Where spherical symmetry has been used to reduce the calculation to integration over r and l alone. Note r is integrated over the whole nuclear volume (to radius R), while l is integrated out to the maximum shell radius which can possibly overlap with the nucleus (radius $2R$). This integration can be carried out in two parts, starting with values which depend on the DSB radial position:

$$\int_0^R V_f 4\pi r^2 dr =$$

$$\int_0^{R-t} 4\pi r^2 dr + \int_{R-t}^{R} \left(\frac{2rl - l^2 - r^2 + R^2}{4rl}\right) 4\pi r^2 dr =$$

$$\frac{4}{3}\pi(R-r)^3 + \pi d\left(\frac{17}{12}l^2 - 4lR - 3R^2\right)$$

Where this integral has been separated according to the changing expressions for of V_f. By dividing through by the $\frac{4}{3}\pi R^3$ term and simplifying we obtain:

$$\frac{1}{\frac{4}{3}\pi R^3}\int_0^R V_f 4\pi r^2 dr = \frac{1}{16}\left(\frac{l}{R}-2\right)^2\left(\frac{l}{R}+4\right)$$

Substituting this into the expression for Θ gives:

$$\Theta = \int_0^{2R} \frac{\left(\frac{l}{R}-2\right)^2\left(\frac{l}{R}+4\right)}{16} e^{-\frac{l^2}{2\sigma^2}} 4\pi l^2 dl$$

This expression can be expanded and integrated analytically to give

$$\Theta(R,\sigma) = \frac{2\pi\sigma^2}{R^3}\left(\sqrt{2\pi}R^3\sigma\,\mathrm{erf}\left(\frac{R\sqrt{2}}{\sigma}\right) - e^{-\frac{4R^2}{2\sigma^2}}(\sigma^4 - R^2\sigma^2) + (\sigma^4 - 3R^2\sigma^2)\right)$$

Where we can now see that this overlap probability depends only on the values of the overlap coefficient σ and the nuclear radius R.

Average Correct Repair Probability

For any given break in a particular condition, its probability of correct repair is given by $\frac{1}{1+\zeta_{tot}}$, where ζ_{tot} is the particular sum of recombination rates seen by that cell. While we have calculated the average rate, $\Theta(R,\sigma)$ above for a single break, in a realistic scenario ζ_{tot} has some distribution about this mean, which must be taken into account to characterise the overall behaviour of the system.

The true distribution of integral rejoining rates is an extremely complex function, due to the interplay of the $e^{-\frac{d^2}{2\sigma^2}}$ term with the distribution of breaks within the cell nucleus, and lacks a simple analytic expression for its overall distribution. However, the overall behaviour is very accurately characterised by a simplifying assumption.

Specifically, it is possible to approximate the distribution of breaks as a number of discrete events, which are Poisson distributed with mean $\rho\Theta(R,\sigma)$. This is equivalent to approximating the recombination function as a step function with a value of 1 out to some maximum radius d_{max} and 0 outside. In this case, the probability of correct repair for a given break simplifies to $\frac{1}{1+n}$, where n is the number of breaks within a distance d_{max} of the break under consideration.

By assuming this distribution is Poisson distributed with mean λ, we can then say that the expectation value that a randomly chosen break will be repaired correctly is given by

$$P_{corr} = \sum_{n=0}^{\infty} \frac{e^{-\lambda}\lambda^n}{n!}\frac{1}{1+n} = e^{-\lambda}\sum_{n=0}^{\infty}\frac{\lambda^n}{(n+1)!}$$

If we let $y = n + 1$, then

$$P_{corr} = \frac{e^{-\lambda}}{\lambda}\sum_{y=1}^{\infty}\frac{\lambda^y}{y!} = \frac{e^{-\lambda}}{\lambda}\left(e^{-\lambda} - 1\right) = \frac{1 - e^{-\lambda}}{\lambda}$$

By fixing the average number of breaks equal to the expected interaction rate $\lambda = \rho\Theta(R, \sigma)$, this can then be used to calculate the total probability of misrepair as a function of number of breaks and recombination radius.

Misrepair Validation

To test the validity of this model, analytic predictions were compared to a simple Monte Carlo model of rejoining. In this simple model, DSBs consisting of pairs of free ends were distributed randomly within a spherical nucleus of radius 1. At each timestep, each pair of free ends was randomly tested for rejoining, with a probability given by $e^{-\frac{d^2}{2\sigma^2}}\Delta t$, where d is the separation between the free ends, and Δt is a timestep chosen to give reasonable rejoining rates. Breaks were then scored as correctly rejoined (two free ends from the same DSB rejoined) or misrejoined (free ends from different DSBs rejoined), to calculate the total rate of misrejoining in different conditions. By carrying out a series of such simulations, the total rate of misrepair was calculated for different combinations of break number and rejoining range σ, and are plotted as points in Supplementary Figure 2.

When analytic predictions are compared to the Monte Carlo values, it can be seen that while the Poisson approximation is accurate when σ is not much smaller than R, at very small σ there is a divergence between the models. This is seen to be the result of the very high degree of skew in the distribution of ζ_{tot} causing the mean to be a less accurate representation of the overall behaviour of the system. To address this, a simple empirical scaling function was applied to the mean value, of the form $\omega(R, \sigma) = A + (1 - A)e^{-\frac{B\sigma}{R}}$. A and B were obtained by fitting the analytic model to the Monte Carlo simulation, with values of A=0.757 and B=5.39 found to effectively account for this skew, with analytic and MC curve coefficients agreeing to within 1%. The resulting curves are plotted as lines alongside the Monte Carlo model in Supplementary Figure 2, of the form

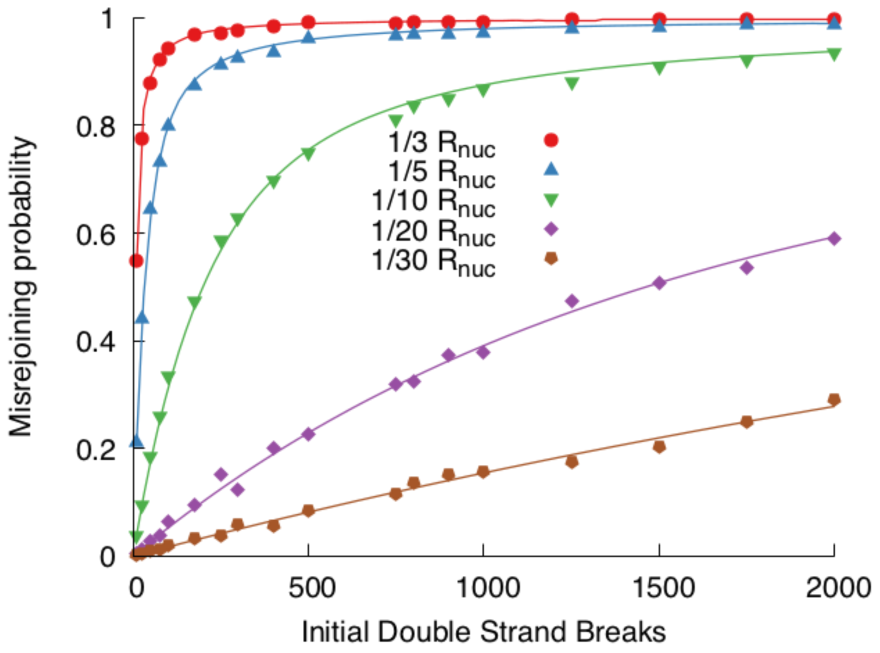

Figure S2. Comparison of Monte Carlo calculations of misrepair rates (points) to analytic calculation, as a function of initial double strand breaks, for different values of σ (legend). As expected, the yield of misrejoining events increases as the number of DSBs increase for all values of σ. The yield of aberration increases rapidly as σ increases, particularly as it becomes comparable in size to the total cell radius.

$$P_{Correct} = \frac{1 - e^{-\eta(N_0,R,\sigma)}}{\eta(N_0,R,\sigma)}$$

where $\eta(N_0, R, \sigma) = \rho\Theta(R,\sigma)\omega(R,\sigma)$ as in the main text, showing good agreement over a wide range of break counts and rejoining coefficients.

Chromosome Aberrations

By assuming chromosomes occupy spherical sub-volumes within the nucleus, inter- and intra-chromosome interaction rates can be calculated identically to the whole-nucleus rates, above. For calculating the total rate of recombination within a chromosome territory, an identical approach to that used to calculate $\Theta(R,\sigma)$ above can be used, but substituting the smaller chromosome radius r_c for R everywhere.

To calculate the recombination rate within some sub-volume of a larger volume, the integral must be performed with appropriate limits. For example, considering the probability of an intra-chromosome recombination at a distance of less than r_D, as a function of rejoining events which occur in the chromosome radius r_c. This can be carried out by altering the integration limits on the calculation of Θ:

$$\Theta(r_c, \sigma, r_D) = \frac{1}{\frac{4}{3}\pi R^3} \int_0^{r_c} \int_0^{r_D} V_f \, e^{-\left(\frac{t^2}{2\sigma^2}\right)} 4\pi t^2 4\pi r^2 dt dr$$

Which can be solved in the same fashion as the whole-nucleus case to give

$$\Theta(R, \sigma, r_c) = \frac{\pi\sigma^2}{4R^3} \left(\begin{array}{c} 8\sqrt{2\pi} R^3 \sigma \operatorname{erf}\left(\frac{r_c\sqrt{2}}{\sigma}\right) \\ -e^{-\frac{r_c{}^2}{2\sigma^2}}\left(r_c^4 + 4r_c^2(\sigma^2 - 3R^2) + 16 r_c R^3 + 8\sigma^2(\sigma^2 - 3R^2)\right) \\ +(8\sigma^4 - 24R^2\sigma^2) \end{array} \right)$$

As noted in the main text.

Model Fitting

As described in the main text, a range of data sets covering DNA repair kinetics and fidelity, chromosome aberration yield and mutation rates were obtained from the literature, and use to parameterise the model. For each experiment, key experimental conditions were extracted – Dose, cell type (genome size & chromosome number), the time point following irradiation, any DNA repair defects present in the cell, the phase in which the irradiation was carried out, and the experimental endpoint (together with the gene size, in the case of mutation rates). These data were then used as input into an implementation of the above model in python to calculate model predictions for each of the experimental conditions. The characteristics of the cell lines used in data considered in this work are presented in Table S2.

The model was fit to the data by varying a set of nine parameters - λ_F, λ_S, λ_M, p_c, p_{fail}, σ, μ_{NHEJ}, μ_{MMEJ} and v – simultaneously in a fit across all data at once. A single set of parameter values was applied to model results from all cell lines and conditions, with variations reflected by mechanistic considerations. This fit was carried out in python using the Scipy curve_fit method. This method uses the Levenberg–Marquardt least squares method to calculate the set of parameters which minimise the difference between model predictions and experimental observations, weighted according to the uncertainty on the observations. Parameter variances are provided by the diagonal elements of the model parameter covariance matrix, with the quoted uncertainties given by their square root.

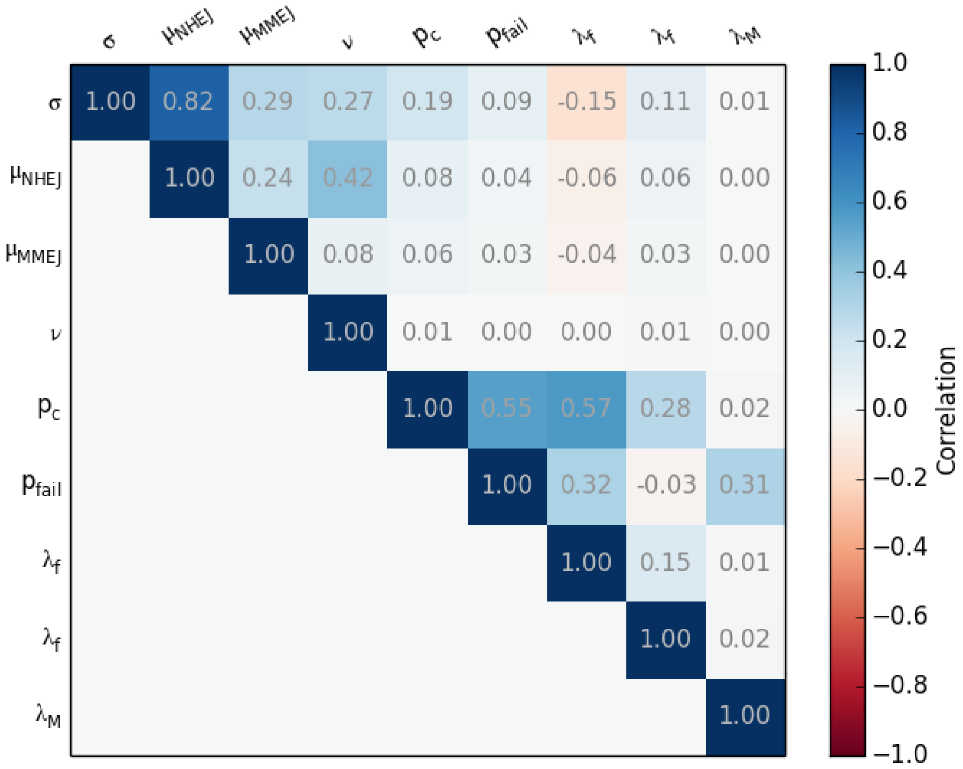

Figure S3. Correlation matrix for best-fitting parameter set. While there is some correlation between fidelity-related parameters (top left) and repair rate parameters (bottom right) values are typically small, and do not dramatically impact on confidence intervals of fitted parameters.

The full fit values and parameters can be found in Table 1 in the main text, showing that parameters are well defined, with reasonable confidence intervals. In addition, because the fit makes use of a range of experimental endpoints, the total covariance between parameters is limited as most parameters or small groups of parameters are strongly limited by specific experimental data sets. The minimum identified also appears to be globally unique, as it is reliably reached by converging fits at a wide range of starting values (tested for initial values randomly distributed within a range from 0.1 to 10x best fitting parameters). The correlation matrix for the best fitting parameter set is illustrated in Supplementary Figure 3. Here it can be seen that there are two groups of parameters which show some covariance (the fidelity-related parameters, in the top left, and the kinetic parameters in the bottom right), but for most points these effects are weak and do not significantly expand the fitting confidence intervals.

Table S2 Summary of cell lines used in experimental data analysed in this work

Cell Line	Species	Tissue	DNA Repair	Mutation	Publications	Figures
C2906	Human	Fibroblast	Normal		Beucher 2009	1
2BN	Human	Fibroblast	NHEJ Defective	XLF deficient	Beucher 2009	1
HSC62	Human	Fibroblast	HR Defective	BRCA2 mutant	Beucher 2009	1
	Mouse	Embryonic fibroblast	Normal		Beucher 2009	1
	Mouse	Embryonic fibroblast	NHEJ Defective	DNA Ligase IV deficient	Beucher 2009	1
	Mouse	Embryonic fibroblast	HR Defective	Rad54 deficient	Beucher 2009	1
MRC-5	Human	Fibroblast	Normal		Lobrich 2000, Kühne 2004	1,2,5
180 BR	Human	Fibroblast	NHEJ Defective	DNA Ligase IV deficient	Kühne 2004, Kasten-Pisula 2005	1,5
GM38	Human	Dermal fibroblast	Normal		Rydberg 2005	2
AG0-1522	Human	Fibroblast	Normal		Cornforth 1987, Corrnforth 2002, Gotoh 2009, Liu 2010	3,5
HF12	Human	Lung fibroblast	Normal		Simpson 1996	3
HF19	Human	Lung fibroblast	Normal		George 2009, Ponomarev 2014	3
M059K	Human	Glioblastoma	Normal		Virsik-Kopp 2003	3
M059J	Human	Glioblastoma	NHEJ Defective	DNA-PK deficient	Virsik-Kopp 2003	3
A_L	Human-Hamster Hybrid	Ovary	Normal		Schmid 2011	3
V79	Hamster	Lung	Normal		Sachs 1997, Belli 2002	3,4,5
CHO	Hamster	Ovary	Normal		Morgan 1990, Hu 1996, Freyer 1997, Schwartz 2000, Rothkamm 2003, Lin 2014	4,5
V3	Hamster	Ovary	NHEJ Defective	DNA-PK deficient	Rothkamm 2003, Lin 2014	5
xrs6	Hamster	Ovary	NHEJ Defective	Ku80 deficient	Freyer 1997, Rothkamm 2003	5
411BR	Human	Fibroblast	NHEJ Defective	DNA Ligase IV deficient	Kühne 2004	5
A2780	Human	Ovarian carcinoma	Normal		Biade 1997	6
HT-29	Human	Colorectal Adenocarcinoma	Normal		Biade 1997, Stobbe 2002	6
OVCAR	Human	Ovarian carcinoma	Normal		Biade 1997	6
U2OS	Human	Osteosarcoma	Normal		Giunta 2010	6
